# Deriving forest fire ignition risk with biogeochemical process modelling^☆^

**DOI:** 10.1016/j.envsoft.2014.01.018

**Published:** 2014-05

**Authors:** C.S. Eastaugh, H. Hasenauer

**Affiliations:** a Institute of Silviculture, Department of Forest and Soil Sciences, Universität für Bodenkultur, Peter-Jordan Str. 82, A-1190 Wien, Austria; b School of Environment, Science and Engineering, Southern Cross University, PO Box 157, Lismore, NSW 2480, Australia

**Keywords:** Wildfire, Fire risk, BIOME-BGC, Fire index, Climate change regions, Risk indices, Ignition

## Abstract

Climate impacts the growth of trees and also affects disturbance regimes such as wildfire frequency. The European Alps have warmed considerably over the past half-century, but incomplete records make it difficult to definitively link alpine wildfire to climate change. Complicating this is the influence of forest composition and fuel loading on fire ignition risk, which is not considered by purely meteorological risk indices. Biogeochemical forest growth models track several variables that may be used as proxies for fire ignition risk. This study assesses the usefulness of the ecophysiological model BIOME-BGC's ‘soil water’ and ‘labile litter carbon’ variables in predicting fire ignition. A brief application case examines historic fire occurrence trends over pre-defined regions of Austria from 1960 to 2008. Results show that summer fire ignition risk is largely a function of low soil moisture, while winter fire ignitions are linked to the mass of volatile litter and atmospheric dryness.

## Highlights

• Models developed for forest productivity studies can be used to predict fire ignition risk.
• Modelled labile carbon levels can be a useful proxy for highly flammable fine fuels.
• The fire regime in Alpine Europe in summer and winter has different climatic drivers.
• An increase in summer extreme fire risk days is predicted.

## 1 Introduction

Climate change is expected to impact forests in a number of ways, both directly and indirectly. One of the most important indirect effects in many regions is the possibility of increasing wildfire risk (Brown et al., 2004), and the introduction of fire as an important shaper of landscapes in areas where this has not been the case for centuries or millennia (Schumacher, 2004). Fires are an integral part of many forest ecologies, and have always been fundamental in shaping forest structures and assemblages (Bond et al., 2005; Bowman, 2005; Lynch et al., 2007). Fire regimes are strongly interlinked with climate changes (Whitlock et al., 2003; Meyer and Pierce, 2003; Taylor and Beaty, 2005), and so it is not unreasonable to expect changes in the occurrence and severity of forest fires in many regions. Increased temperatures alone do not necessarily mean that more fires will occur; several other climatic and non-climatic factors are also involved such as ignition sources, fuel loads, vegetation characteristics, rainfall, humidity, wind, topography, landscape fragmentation and management policies (Flannigan et al., 2000). Taking these factors into account Flannigan et al. (2005) reviewed fire predictions for North America and suggested that overall increases in area burned may be in the order of 74–118% by the end of the 21st century. A further observed impact of recent environmental change is an increase in forest growth in many areas (i.e. Phillips et al., 1998; Hasenauer et al., 1999; Nemani et al., 2003), which may lead to increased fire hazard due to changing fuel loads (Lenihan et al., 1998) and depleted soil moisture.

Several studies have been conducted in an attempt to assess possible future fire risk under changed climatic conditions (i.e. Pitman et al., 2007; Malevskii-Malevich et al., 2007; Good et al., 2008; Krawchuk et al., 2009; Holsten et al., 2013). Well-validated predictive tools for forest fire risk would be useful for resource allocation (Cantwell, 1974; McCarthy et al., 2003; Prestemon and Donovan, 2008), emergency services budgeting (Thompson et al., 2013), infrastructure planning (Eastaugh and Molina, 2011, 2012) and warning systems (Valese et al., 2010; Arpaci et al., 2013). Such studies generally rely on defining some meteorological index of fire risk, and calculating the development of that index under future climate scenarios. Inherent in this approach is the assumption that the relationship between the meteorological variables and fire risk will remain constant, which may not be the case if the future climate, management activities or natural ecosystem development with aging alters forest conditions. Recent work from Pausas and Paula (2012) suggests that ecosystem productivity may be a key determinant of fire sensitivity, in which case an ideal fire risk indicator must take such non-climatic factors into account.

Numerous fire risk indices have been developed over the past seventy years, beginning with the purely empirical meteorological indices of Ångström and Nesterov in the 1940s. Käse (1969) modified the Nesterov index to account for higher probability of fire in spring, dependent on the budburst date of birch and robinia trees. Keetch and Byram (1968) developed an index of soil moisture deficit (KBDI) for use by fire agencies, on the principle that soil dryness is likely to be accompanied by fuel dryness. The more sophisticated Canadian Forest Fire Weather Index of Van Wagner (1987) expressly considers how weather conditions affect the moisture content of different fire fuel layers. The more easily ignited fine fuels lose moisture quickly under dry atmospheric conditions, while larger fuels dry only after extended periods. Tanskanen and Venäläinen (2008) have pointed out however that the accuracy of the indices can vary seasonally depending on the proportion of dead to live fine fuels on the forest floor. To some extent this suggests that fire ignition risk may potentially be reduced through appropriate management regimes, although this will require a comprehensive understanding of how ignition risk relates to forest physiological processes. It is likely that the sophisticated biogeochemical process models developed over recent years may be useful to assist this understanding. We chose here the Angström, Nesterov and KBDI indices to represent a continuum of fire index types from a simple instantaneous index, to one that includes consideration of precipitation, to a more complex accumulative index with a physical interpretation.

Many of the various parameters implicated in forest fire behaviour are also important parameters in biogeochemical forest growth models. Keane et al. (1996) took advantage of this in linking the FOREST-BGC model of Running and Coughlan (1988) with the specifically fire-optimised gap model FIRESUM (Keane et al., 1989). More recently, Keane et al. (2011) used the BIOME-BGC model (Thornton, 1998) as an input into Fire-BGCv2, a highly sophisticated research tool linking biogeochemical modelling, forest succession models and fire spread behaviour. Management-sensitive parameters such as fuel volumes clearly have an impact on fire behaviour (Finney, 2006), but the possible link between these parameters and the relative likelihood of fire ignition has not yet to our knowledge been explicitly studied with a biogeochemical model.

Biogeochemical models track the pools and fluxes of water, carbon and (often) nitrogen in an ecosystem, and allocate the carbon taken up by photosynthesis to various components of the system. With various degrees of complexity, the models include consideration of soil moisture and litter volumes and composition. Our contention is that these variables may prove to be reasonable indicators of forest fire ignition risk, at least comparable with meteorological indices over short time frames. If this is the case, then it is likely that the model-derived indices would be superior over longer timescales, due to their incorporation of how forests change over time, particularly in a changing climate or under varying forest management practices.

Although the focus of this work is on exploring the use of biogeochemical forest growth modelling in forest fire risk evaluation, we also provide a brief application case, examining the seasonal drivers and trends of fire ignition risk in Austria. Central European alpine regions are not typically considered high fire risk areas but there is mounting concern over the possibility of increased risk in the near future (Conedera et al., 2006; Gossow et al., 2009; Wastl et al., 2012). Fires are generally not large, but in rugged terrain they can be difficult to control and can have serious long term effects on the protection function of mountain forests (Brang et al., 2006; Sass et al., 2010, 2012a,b). Conedera et al. (1996) reported an increase in forest fires in Switzerland from the 1960s and 1970s, and noted that this “could not be explained simply through the analysis of particular meteorological factors or the inclusion of the major anthropogenic causes”, while Zumbrunnen et al. (2009) have pointed to the importance of both meteorological and fuel load conditions to fire occurrence in Alpine areas. We choose the Austrian situation due to the availability of a quality-checked national fire database (Müller et al., 2013; Eastaugh and Vacik, 2012), a validated climate interpolation (Thornton et al., 1997; Petritsch and Hasenauer, 2007) and a previously parameterized biogeochemical model (Pietsch et al., 2005; Eastaugh et al., 2011).

The purpose of this study is to assess whether the inclusion of forest physiological properties can potentially provide improved estimates of forest fire ignition risk, compared to simple meteorological indices. We apply the species-specific version of BIOME-BGC (Pietsch et al., 2005) to 2014 forested sites of the Austrian National Forest Inventory (NFI; Gabler and Schadauer, 2006), and record daily values of simulated soil water content (*sw*), labile litter carbon (*llc*) and vapour pressure deficit (*vpd*) at each site from 1960 to 2008. Defining two indices as BGC-SW = *sw* and BGC-LV as llc*vpd, we assess their precision against recorded fire occurrence data from 1995 to 2008, and compare this with the precision of the Angström, Nesterov and KBDI indices. For the application study the model outputs are geographically aggregated according to regions defined by Eastaugh et al. (2011), being those parts of Austria that have experienced climate change over the past half-century of more than one standard deviation different to the national average. The output of the application study shows trends in the BGC-LV index from 1960 to 2008, and compares the BGC-SW extremes from 1991 to 2008 against a 1960–1990 baseline. The model and the index performance comparisons allow us to suggest explanations for the seasonal variation in forest fire risk in Alpine areas. Specific outputs are:

a) A comparative evaluation of the five indices at the national scale, for both summer and winter seasons, in terms of their ability to reflect overall fire ignition risk and the occurrence of extreme risk conditions,
b) An analysis of overall trends in fire ignition risk at the regional scale using the BGC-LV index, and
c) An analysis of trends in extreme summer fire ignition risk at the regional scale using the BGC-SW index.

## 2 Technical background

### 2.1 Meteorological indices

#### 2.1.1 Angström

Ångström (1942, cited by Ångström, 1949) developed a simple instantaneous meteorological index relating fire risk to relative humidity (*Rh*) and temperature (*T*) (Eq (1)) from field experiments in Sweden. The Angström index *AI* is calculated as:

(1) $$AI=\frac{Rh}{20}+\frac{29-T}{10}$$

The *AI* gives lower values when fire risk is higher. Fire is generally considered ‘very likely’ at values less than 2.0, and ‘unlikely’ at values over 4.0.

#### 2.1.2 Nesterov

Nesterov (1949) constructed a cumulative index, where each day's calculated value is added to that from the previous day. Each daily increment is calculated as:

(2) $$N_{d}=T_{\max}\left(T_{\max}-T_{\text{dew}}\right)$$

where *T*_max_ and *T*_dew_ are the daily maximum and dew-point temperatures respectively. We estimate *T*_dew_ as being equal to the daily minimum temperature *T*_min_, an approximation that has been shown to be accurate except under arid conditions (Kimball et al., 1997a). The summation continues until such time as some minimum amount of rainfall is experienced: in Austria 4 mm is used (Arpaci et al., 2010). The accumulated Nesterov index value *NI* then is:

(3) $$NI=\sum_{i=1}^{w}N_{d_{i}}$$

where *W* is the number of days since 4 mm of rainfall was collected. The Nesterov index is currently used as an official indicator of fire risk in Russia (McRae et al., 2006) and in Austria (Arpaci et al., 2010). The index is interpreted with the aid of the threshold values in Table 1.

#### 2.1.3 Keetch–Byram drought index

The Keetch–Byram drought index (KBDI; Keetch and Byram, 1968, typographical errors corrected 1988) was specifically designed as a fire weather warning system. We use here the metric version presented by Crane (1982, cited by Alexander, 1990).

(4) $$dK=10^{-3}\frac{\left(203.2-{\text{KBDI}}_{t-1}^{*}\right)\left(0.968{\text{e}}^{\left(0.0875T_{\max}+1.5552\right)}-8.3\right)}{1+10.88{\text{e}}^{-0.001736R_{\text{av}}}}$$

where ${\text{KBDI}}_{t-1}^{*}$ = the previous day's moisture deficiency less the net rainfall; *R*_av_ = local annual average precipitation; *dK* = the daily addition to moisture deficiency.

The KBDI increases daily according to temperature and humidity, and decreases by the net rainfall when rainfall *P* over consecutive days exceeds 5.1 mm. Net rainfall is the depth of the rainfall minus the 5.1 mm buffer. After Janis et al. (2002):

$$\begin{array}{l} {\text{KBDI}}_{t}={\text{KBDI}}_{t-1}+dK\;\text{if}\;P_{t}=0\\ {\text{KBDI}}_{t}={\text{KBDI}}_{t-1}+dK\;\text{if}\;P_{t}>0\;\text{and}\;\sum P=5.1\;\text{mm}\\ {\text{KBDI}}_{t}={\text{KBDI}}_{t-1}^{*}+dK\;\text{if}\;P_{t}>0\;\text{and}\;\sum P>5.1\;\text{mm} \end{array}$$

The KBDI is intended as a direct indicator of soil water deficit. In its original form, it estimated the amount of net precipitation in points (1 point = 1/100th of an inch = 0.254 mm) necessary to bring the soil to saturation. In the metric form the index is measured in millimetres, and ranges from zero (saturated soil) to a maximum of 203.2 mm. Keetch and Byram (1968) divided the index into eight ‘stages’, but point out that the significance of each stage will depend on local climatological conditions. Geographical variations in KBDI across Austria were studied by Petritsch and Hasenauer (2014).

### 2.2 BIOME-BGC

We use here a version of the BIOME-BGC model modified and calibrated for central European conditions (Pietsch et al., 2005; Pietsch and Hasenauer, 2006). Storages and fluxes of water, carbon and nitrogen are tracked throughout various pools in the vegetation, litter and soil on a daily timestep. Ecological processes in the model are driven by daily meteorological data, site characteristics and various ecophysiological parameters describing the vegetation at each site (see Table S1 in the Supplementary Information). The model is not specifically calibrated for this study, and is run with standard settings, parameterised by Pietsch et al. (2005) for Norway spruce (*Picea abies*), Scots pine (*Pinus sylvestris*), Beech (*Fagus sylvatica*), Larch (*Larix decidua*), Oak (*Quercus robur* and *Q. petraea*) and Stone pine (*Pinus cembra*). Norway spruce is parameterised separately for high and low elevation sites. Assumptions of atmospheric CO_2_ follow the IS92a curves (IPCC, 1992), and atmospheric nitrogen deposition is modelled for each NFI point according to Eastaugh et al. (2011). Soil depth and composition are interpolated from the Austrian National Forest Soil Survey (Englisch et al., 1992) by Petritsch and Hasenauer (2007) using the Kriging method.

This model version was also successfully applied to the sample points of the Austrian NFI by Huber et al. (2013). The model has been comprehensively described elsewhere, so we refer the reader to Thornton et al. (2002), Eastaugh et al. (2011) and the papers cited in this paragraph for detailed technical descriptions of the model's operation. In brief, carbon is simulated as entering the ecosystem via photosynthesis, and lost through autotrophic respiration. The remaining net primary production is assigned to various vegetation pools as biomass. Biomass can be reduced through management activities (resulting in removal from the system or transfer to the detrital pools), through mortality or through leaf senescence and litterfall. Leaf area index (LAI) is an internally calculated variable and controls canopy radiation absorption, light transmission to the ground and precipitation interception. The litter input to the detrital pools also varies according to LAI. Atmospheric nitrogen is input to the soil via the stomatal uptake and detrital processes, and the model limits the amount of nitrogen available for plant growth depending on microbial demands in the soil, which in turn depend on the mass of litter, the temperature and the soil moisture sensitive decomposition rates. Forest management (thinning) in BIOME-BGC is simulated through the removal of carbon and nitrogen from the living biomass pools. Part of this is removed from the system (assumed to be forest products), and part is reassigned to the coarse woody debris and litter pools. These proportions and the timing of the interventions are user-defined; for this study we used the expert interview-based assumptions of Petritsch (2008).

The model includes four separate pools representing carbon in litter: labile carbon, two pools of cellulose carbon (depending on whether bound by lignins or unbound) and lignin carbon. Litterfall in coniferous forests is assumed to be constant for each day of the year (Pedersen and Bille-Hansen, 1999), with the daily rate reset each year depending the previous year's maximum LAI and a species-specific turnover rate. Breakdown of litter depends on the simulated action of soil microbes, which is highly temperature dependent. Labile carbon pools break down quickly (by definition), and as a result peak in the late winter/early spring season, as it has accumulated over the winter when conditions do not support microbial degradation. After this time breakdown is swift in the warming soils of late spring and summer.

Our proposition in this work is that the mass of carbon in the labile litter carbon pool (*llc*) can be used as a proxy for the volume and ignitability of highly flammable surface litter, which in coniferous forests contains high levels of volatile compounds (Ormeño et al., 2009; Zhao et al., 2012). Fine dead fuels dry quickly (Nelson, 2001; Wotton, 2009), and as a direct measure of the drying ability of the atmosphere we use the vapour pressure deficit (*vpd*). Our first model-derived index is thus:

(5) $$\text{BGC}-\text{LV}=llc*vpd*10^{3}$$

where *llc* is the modelled mass of labile litter carbon in kg/m^2^ and *vpd* is the vapour pressure deficit in Pa. Higher values indicate a greater mass of volatile litter and/or greater vapour pressure deficit, and thus higher fire ignition risk.

BIOME-BGC focuses strongly on water cycling, and has been extensively used in hydrological studies (i.e. Kimball et al., 1997a; Pietsch et al., 2003; Warren et al., 2011; Peng et al., 2013). Precipitation may be intercepted by the canopy or entered into the soil water (*sw*) pool for either storage, deep drainage or overland flow. Snowpack is represented by a separate pool, with fluxes according to precipitation at temperatures below 0 °C, melting at temperatures over 0 °C and sublimation according to solar radiation. Soil water loss through evapotranspiration is calculated with the Penman–Monteith equation as a function of air temperature, air pressure, *vpd*, incident solar radiation and the transport resistance of water vapour and sensible heat. Evapotranspiration depends on both climatic and physiological characteristics of leaf area and species-specific parameters and thus is strongly linked to stand conditions.

Soil moisture can often be a useful proxy for surface fuel moisture (Keetch and Byram, 1968; Hatton et al., 1988), so our second model-derived index is simply the equivalent depth of soil water in mm:

(6) BGC-SW=sw

Higher values of the BGC-SW index indicate greater soil moisture content and thus lower fire ignition risk.

### 2.3 A note on terminology

Wildfire research is often made more complicated through confusions between the terms ‘danger’, ‘hazard’ and ‘risk’ (Bachman and Allgower, 2001). Although there is not yet any definitive agreement on terminology, Hardy (2005) is clear that ‘hazard’ relates primarily to fuel conditions, and must always be independent of weather. Bachman and Allgower (2001) dismiss the term ‘fire danger’ as being “*…defined by human and societal perceptions…*” and thus “*… useless for wildland fire research and management*.” In risk engineering the concept of ‘risk’ includes components of both the likelihood of an event occurring and the consequences of that event, although in wildfire research the consequences are often considered separately from the event probability, leading to Hardy's (2005) definition of ‘Fire risk’ as “ *– the chance that a fire might start, as affected by the nature and incidence of causative agents*.” In this paper we are concerned with the relative probability of fire ignitions occurring, dependent on climatic and environmental factors. We do not consider the source of the ignition, or consequences beyond the fact that the resulting fire was sufficiently persistent to demand response from fire agencies and hence entry into the database. For the purposes of this paper we can define this as ‘ignition risk’.

## 3 Data

Austria is a landlocked country of 83 855 km^2^ in Central Europe, with a predominantly mountainous terrain and around 47.6% forest cover. Forests are mostly coniferous, with 50.7% Norway spruce (*Picea abies*), 10.0% Beech (*Fagus sylvatica*) and 5.1% *Pinus* spp., mostly Scots pine (*P. sylvestris*) (Quadt et al., 2013). Climate is geographically variable, ranging from Pannonian in the east and Atlantic in the west to Alpine in the high mountains.

### 3.1 Climate

Daily climate data is required to calculate fire index values and also act as drivers for the BIOME-BGC model. The Austrian National Forest Inventory is arranged as clusters of four points on the corners of a 200 metre square, with clusters arranged over the country on a square grid of 3.89 km. We interpolate daily climate for the southeast point of each cluster with the DAYMET model of Thornton et al. (1997), as adjusted and validated by Hasenauer et al. (2003). DAYMET interpolations are generated based on the geographic position, elevation, slope, aspect and angle to the horizon and climate records from several hundred climate stations in Austria and surrounding countries. Daily meteorological data at such high geographic density is limited to temperature and precipitation records, so secondary variables must be derived from these measures. DAYMET directly interpolates precipitation and maximum and minimum temperature, and from this is possible to calculate mean daily temperature, growing season length, vapour pressure deficit and solar radiation. Solar radiation and water vapour pressure deficit values are derived from daily temperature and precipitation according to the methods of Thornton et al. (2000), validated for Austria by Hasenauer et al. (2003), and incorporate the potential shadowing effect of surrounding mountains and the radiation reflections due to snowpack. The DAYMET output thus provides all the necessary information to calculate daily values of the indices for each NFI point, and forms the climatic input for the BIOME-BGC model.

**Fig. 1:**
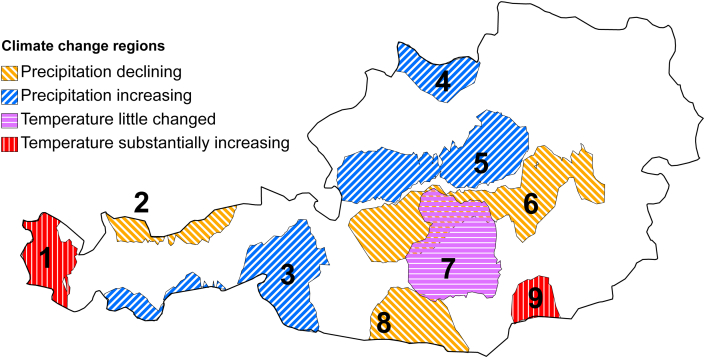
Climate change regions (adapted from Eastaugh et al., 2011).

Overall, Austrian forests have experienced an average warming of 1.5 °C over the past fifty years, with no discernible trend in precipitation (Eastaugh et al., 2010). There is however marked sub-national variability and Eastaugh et al. (2011) delineated nine ‘climate change regions’ where temperature or precipitation trends were more than one standard deviation outside the national average (Fig. 1).

### 3.2 Austrian fire database

As part of the ALP FFIRS project (Valese et al., 2010) a database of Austrian wildfires has been compiled based on historic documentary records (Vacik et al., 2011; Müller et al., 2013). The database has been assembled with information from a variety of sources that cover different time periods or geographical regions. The database contains records of 1035 forest fires between 1995 and 2008 and is used to assess the precision of each of the fire ignition risk indices at the national scale, separately for summer and winter conditions. Data for periods prior to this is likely to be incomplete (Eastaugh and Vacik, 2012), so purely data-based analyses of long-term trends are impossible. Areas burnt are not recorded for all ignitions; those that are recorded range from 1 m^2^ to 120 ha. Numbers of ignitions per year range from 7 in 1997 to 226 in 2007. The monthly distribution of fire ignitions is shown in Fig. S1 in the supplementary information.

## 4 Analysis methods

Daily values for all indices are calculated for each forested sample point of the Austrian NFI, according to the equations above. For the index comparisons the daily values are spatially aggregated nationally (using the mean of each daily index), separately for the summer (May to November, 215 days) and winter (December to April, 150 days). These cut-off dates were selected to be in line with work on Alpine forest fires from Austria (Arpaci et al., 2013), neighbouring Switzerland (Conedera et al., 2006) and north-eastern Italy (Valese, 2009), all of which suggest that there may be two distinct fire seasons in alpine Central Europe. This gives two datasets (summer and winter) containing National daily mean values of each index. For trend comparisons we aggregate values separately for each of the numbered regions in Fig. 1, and for the unnumbered ‘0’ region. This gives sets of daily mean index values for each climate change region. The methods are chosen specifically in order to assess the precision of the BIOME-BGC indices in both summer and winter conditions, and allow conclusions to be drawn as to why each index performs better or worse in each season.

### 4.1 Index comparison/validation methods

Indices that quantify fire ignition risk are akin to models in that they are a mathematical simplification of complex ecosystem processes or attributes. The output of these indices is a relative indication of the probability of fire ignition. This is not something that can be directly measured in the field (Schneider et al., 2008), so in the strictest sense ‘validation’ of the indices is not possible. The way to evaluate the effectiveness of these indices then relies on a statistical comparison of index values with the occurrence of recorded fires. In most literature on the topic, this process is referred to as index validation.

#### 4.1.1 Overall hazard

**Fig. S3:**
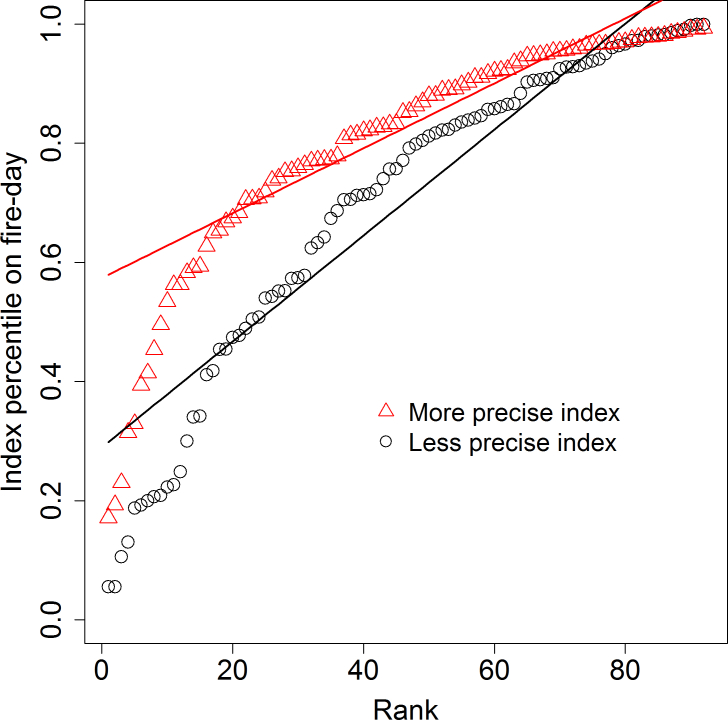
Example figure for index comparison method. For 100 fires, the percentile of the index on each fire day is plotted in rank order. The shape of the curve describes the precision of the index at any percentile, and may be summarized by the intercept and slope of a robust regression line (Eastaugh et al., 2012). The intercept and slope are the skill scores of the index.

Fire indices should be expected to reflect the increase in fire ignition risk as climatic (or other) conditions worsen. This is not necessarily a linear relationship, and various indices have different frequency occurrence distributions over a year or series of years (Fig. S2 in the supplementary information). The shortcomings of using parametric methods of index performance comparison were pointed out by Eastaugh et al. (2012), who suggested a graphical percentile-based technique that we briefly reiterate here. The percentile of each index is calculated for each day in the period of interest, and the percentile values on days when a fire ignition is noted in the database are plotted in rank order. The main characteristics of the resulting curve of points can be described by the intercept and slope of a robust regression line. A hypothetically ‘perfect’ fire index would have its highest values only on days when a fire truly occurs, and thus would plot as a line with an intercept of 100 and a slope of 0, whereas an index of random numbers would approach an intercept of zero and a slope of 100 divided by the total number of fires. Indices are compared at the national scale separately for summer and winter. An example figure demonstrating the method is provided in the supplementary information (Fig S3).

#### 4.1.2 Extremes

For index comparison we define ‘extreme’ fire risk in terms relative to each season, as that percentile of the index that on average would have a 50% chance of exceedence in each season (the 99.67th percentile in winter and the 99.77th in summer). The relative strength of each index at correctly determining extreme fire risk is assessed by the number of fires that did occur at over this percentile value. In terms of the ranked percentile plot, it would be seen as the number of fires above a horizontal line at that particular percentile value.

### 4.2 Trend comparisons

Having tested the usefulness of the BIOME-BGC-based indices, we apply them to each of the 9 climate change regions in Austria and the ‘0’ region that has exhibited change close to the national average (Eastaugh et al., 2011). This section of the work does not use the fire database, but tracks the progress of the indicators from 1960 to 2008. We compare the regions for their trends in both general fire risk and for the occurrence of extremes.

#### 4.2.1 Overall risk

At the National scale and using all climate data from 1960 to 2008 we determine the percentile values of the Nesterov index that correspond to each of the risk classes in Table 1. The BGC-LV index values at these percentiles are presented in a similar table. For each region in each season the trend of each BGC-LV class is estimated with a linear regression of the number of days in each class, in each year.

#### 4.2.2 Summer extremes

Our earlier defined extreme percentiles for soil moisture deficit proved to be too high for trend assessment, as in most regions this level was only exceeded in the drought year of 2003. In order to make meaningful comparisons we chose a percentile of 99.07 (on average, an expectation of 2 days per summer). We divide the database into two time periods (1960–1990 and 1991–2008), and determine the average proportion of years in each time period that experienced more than 2 days of risk above the given percentiles. We then express the change in extreme summer risk (per region) as the percentage change between periods. To illustrate, if in some region prior to 1991 there were 3 years that experienced more than 2 days of extreme fire risk conditions and post 1990 there were 5 years, then ((5/18) − (3/31))/(3/31)*100 = an 187% increase in extreme risk.

## 5 Results

### 5.1 Index comparison/validation

**Fig. 2:**
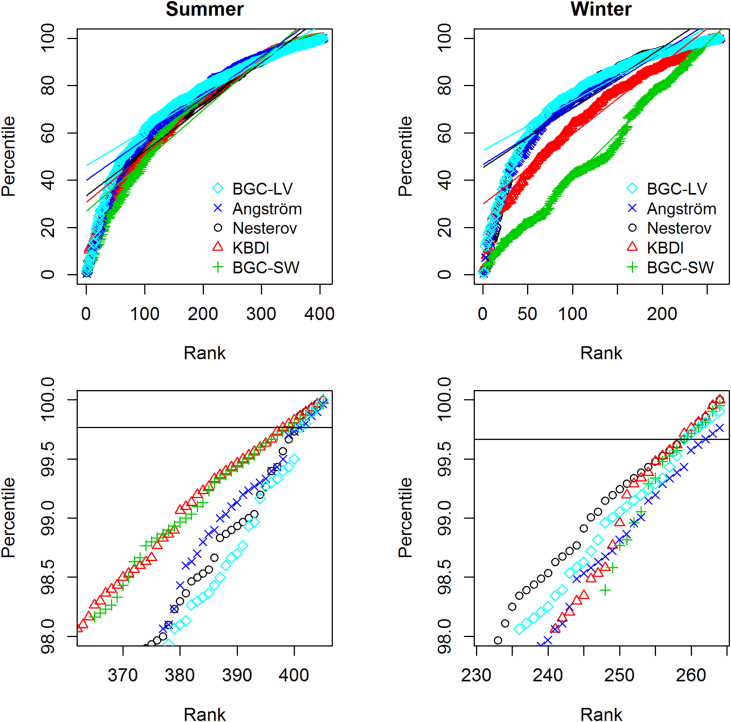
Ranked percentile curves of summer (left) and winter (right) fires. Lower panels are a closeup view of the extreme high values in the upper panels. The superiority of the soil-based indicators in extreme summer conditions is clear in the lower left panel, as is their comparatively poor overall performance in winter in the upper right. For clarity, the order of the indices in the legend matches the order of their ‘*y*’ intercepts in the upper panels.

As an overall descriptor of the seasonal fire ignition risk, the BGC-LV index is superior in both seasons (Table 2). BGC-SW performs poorly as an overall predictor of fires particularly in the winter, but is equal to the KBDI in more extreme conditions in winter and only slightly less precise at the given percentile in summer. Both soil-based indices (BGC-SW and the KBDI) are particularly poor descriptors of overall risk in the winter (Fig. 2, upper right panel) but perform well at extreme values in the summer (Fig. 2 lower left panel).

Based on the encouraging performance of the BGC based indices, trend analyses are performed using the BGC-LV index for an overall assessment of ignition risk, and BGC-SW for summer extremes.

### 5.2 Trends

Class limits for the BGC-LV index were determined as per Table 3. The Nesterov index did not reach class 5 when averaged over the whole of Austria, so the BGC-LV value of 40 was chosen as a suitably rare event to define class 5 in BGC-LV.

**Fig. 3:**
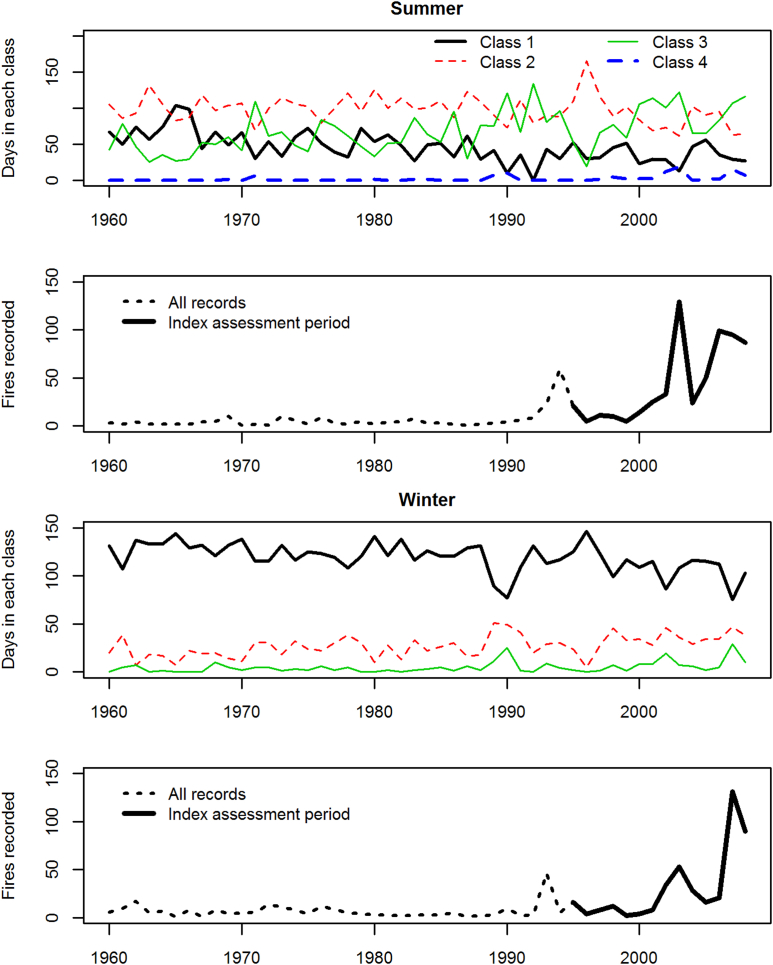
Top and third sub-figures show trends in national average BGC-LV class occurrence. Nationally aggregated, class 5 does not occur in summer, and neither classes 4 nor 5 in winter. Second and bottom sub-figures show the course of recorded fire ignitions.

Fig. 3 shows the nationally aggregated trends in each of the BGC-LV classes. Of interest here is the decline in the ‘no fire risk’ class, in both summer and winter. All individual regions show a decreasing trend of Class 1 and 2 days in summer (Table 4), and an increase in Classes 3 and 4. In winter (Table 5) Class 1 also reduces, but increases are found in Classes 2 and 3.

**Fig. 4:**
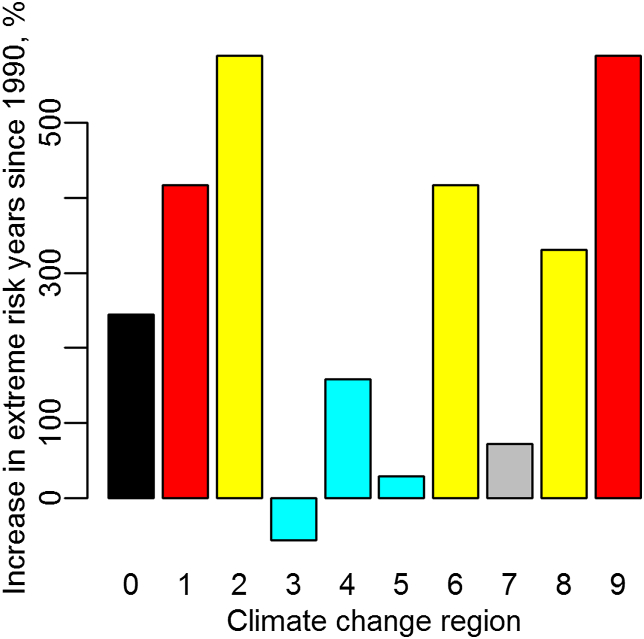
Increase in summer extremes. Columns represent the percentage increase in extreme summer fire danger days for the period 1991–2008, compared to a 1960–1990 baseline. Regions 1 and 9 have had warming trends greater than the national average; region 7 has had no significant temperature change. Regions 3, 4 and 5 have shown increasing precipitation trends while regions 2, 6, and 8 have been drying. Region 0 represents the national average.

Fig. 4 compares the occurrence of summer extreme values of the BGC-SW index in the 1991–2008 period with a 1960–1990 baseline, expressed as a percentage increase in yearly probability. Regions with more warming or drying climate trends are shown to have substantially greater increases in fire risk than the national average.

## 6 Discussion

In principle, simulation models such as BIOME-BGC should be able to provide a more precise indication of fire ignition risk than purely meteorological indices. Pausas and Paula (2012) have recently pointed out the strong links between forest productivity, fuel conditions and fire risk, and recently developed empirical risk models for wildfire in Portugal (Botequim et al., 2013) include the diverse effects of silvicultural treatments. Biogeochemical process models that are optimized for the simulation of forest productivity can thus clearly have a role to play in forest fire science. Although this study has focused on a relatively small region with particular geographic and climatic conditions, the BIOME-BGC model itself has been successfully applied to a wide range of diverse ecotypes around the globe, i.e. tropical forests in South America and Africa (Ichii et al., 2007; Guatam and Pietsch, 2012), boreal forests in North America and Asia (Kimball et al., 1997b; Ueyama et al., 2010), the arctic (Engstrom and Hope, 2011), European and Asian grasslands (Hidy et al., 2012; Han et al., 2013), mangroves (Luo et al., 2010), croplands (Newlands et al., 2012) and countless applications to temperate forests. The dryness and volume of flammable material (particularly fine fuels) have significant influences on fire risk in any fireprone area, and if these can be successfully tracked with a biogeochemical model then it is likely that indices similar to ours could be developed for any region.

A complex biogeochemical model such as BIOME-BGC naturally requires much more input information than simple meteorological indices, and is much more time consuming to use. While at present this would preclude our model from being used operationally by fire agencies, we have shown in this paper that sufficient data exists to run the model on over two thousand sites across Austria and extract information relevant to wildfire studies. The primary advantage that Austria has in this context is the existence of a long-running, detailed, fixed-plot National Forest Inventory. Following Petritsch and Hasenauer's (2014) work on real-time climate interpolation the potential exists for a model such as BIOME-BGC to be maintained on a daily basis, providing an invaluable tool in many areas of environmental research and management.

The BIOME-BGC derived indices in this study are seen to perform well in comparison to the simple meteorological alternatives. The BGC-LV index is a more precise indicator of overall fire risk in both summer and winter, and performs equally as well as the others in extreme winter conditions. Summer extremes however are best represented by the soil based indices, BGC-SW and the KBDI. Although Table 2 suggests that these indices also perform well for winter extremes, the 99.67th percentile chosen for the comparison proved to be too rare an event to make meaningful comparisons of trends. Reducing the threshold to a level where it was exceeded at least once in each region both before and after 1990 brought it down to 98% in the winter, which suggests that winter extremes are localized events and aggregation at the regional scale is not appropriate. Extreme conditions in summer were assessed at the 99.07th percentile, at which level Fig. 2 shows the clear superiority of the soil-based indices (lower left panel). The overall strength of the BGC-LV index for overall ignition risk supports our proposition that volatile fine fuels may be represented by the BIOME-BGC model's labile litter carbon pool, and if modified by the drying power of the atmosphere it is a better overall indicator of fire risk than the Nesterov index. It is possible that in some areas other meteorological indices may offer better performance than those presented here, assuming that the necessary input data is available. As this study is primarily concerned with assessing the performance of the model based indices however, we leave that work to others (i.e. Arpaci et al., 2013). Many more complex meteorological indices depend on windspeed as an input, which limits their application to the calculation of index values at the location of sophisticated weather stations (the approach taken by Arpaci et al., 2013). In complex terrain wind velocities cannot be successfully interpolated in way that temperature and precipitation are, which precluded their use in this study.

Conedera et al. (2006) noted the prevalence of summer fires in inner alpine valleys in Switzerland, and attributed this primarily to the higher levels of lightning activity in this period. Müller et al. (2013) show that in Austria winter fires are rarely lightening caused. Our work here does not deal with ignition causes, but we suggest that the high level of volatile fuels and commonly very high vapour pressure deficits will increase fire risk regardless of the ignition source, as the forest floor becomes more susceptible to fires beginning. Even though most fires in Austria are human-caused (Arndt et al., 2013), the environmental conditions much be such that the dropped cigarette or wayward spark lands on fuels with sufficient ignitability for a fire to begin.

Winter fire risk overall is poorly represented by the soil based indices, strongly suggesting that short-term dryness (likely due to highly localized foehn conditions, Sharples et al., 2010) and fine fuel volatility are the key drivers of the risk. In contrast, in summer the extreme risk days are better represented by soil moisture indices; while the overall summer ignition risk is better assessed with the BGC-LV index at extreme values the soil-based indices are superior, suggesting that long-term drought conditions become the key driver of the risk in extreme summer conditions. This is consistent with the thesis that the availability of highly volatile fine fuels is decreased by microbial activity as summer progresses, and so coarser, slower drying fuels must be ignited for fire to occur. This effect is likely to be particularly pronounced in regions with high fuel buildup under winter snow and extremely dry foehn weather conditions in late winter–early spring.

The assessment of ignition risk trends in this work suggests that changes in extreme conditions in summer may be related closely to climate trends (Fig. 4), although the ‘wetting’ regions 4 and 5 still show an increase in risk above the 1960–1990 baseline (albeit less than region 0). This can be explained by the acceleration in forest growth rates in Austria over recent decades (Hasenauer et al., 1999; Eastaugh et al., 2011). Forest growth trends in Austria in the last mentioned study were found to vary between regions, with warming and wetting regions experiencing greater growth increases, both in observed inventory data and in BIOME-BGC simulations. Faster growing forests must extract more water from the soil, and the strength of the soil-based indices in Fig. 2 shows the clear link between extreme soil dryness and extreme fire risk. What is apparent is an increasing extreme summer fire risk nationally, most clear in warming and drying regions. Only one region (3) has reducing risk, due to increasing precipitation. Other wetting regions (4,5) and the non-warming region (7) have less risk increase that the national average.

The difference between trends in overall ignition risk in Tables 4 and 5 is less easy to interpret, and it may be that the BGC-LV index is weakened by its lack of a cumulative fuel moisture proxy. The Nesterov, KBDI and BGC-SW indices all include consideration of weather conditions prior to the day of index calculation, and thus are more sensitive to the drying of heavier fuels than the Angström or the BGC-LV. It seems likely that some combination of the BGC-LV and BGC-SW indices would be able to capture fire risk under both short and long term drying conditions, but this would doubtless require region-specific parameterization and is well beyond the scope of this initial exploratory study. In an ideal world we would have sufficient data to match modelled risk trends in each region against observed fire occurrence data, but as the length of reliable records is short (Valese et al., 2011; Eastaugh and Vacik, 2012), this is not yet possible in Austria.

It is important to note that the BIOME-BGC model in this study was not specifically calibrated for either soil water or litter levels, but was run under standard assumptions used previously for site-specific calibration (Pietsch et al., 2005) and national-scale forest productivity assessments (Eastaugh et al., 2011). Optimising the model specifically for fire risk purposes would undoubtedly increase its precision. Nevertheless, this initial exploratory study shows the potential for biogeochemical modelling to add to understanding of how climate changes may impact fire risks in forests, and opens a new and exciting direction of practical research.

## 7 Conclusion

Variables tracked in the BIOME-BGC model have proven to be able to track forest fire ignition risk in Austria at precisions comparable to the currently used purely meteorological Nesterov index. In principle, the fact that BIOME-BGC variables are sensitive to both climatic and non-climatic influences suggests that it should be a better indicator of long-term risk trends than solely climate-based indices. The hypothesis that BIOME-BGC's labile litter pool could be used as a proxy for the seasonal buildup of highly flammable fuels has proven reasonable, and in combination with vapour pressure deficit the results are an improvement on common existing risk indices. In extreme summer conditions BIOME-BGC's soil moisture variable closely matches the Keetch–Byram index of soil drought, and has the advantage of being sensitive to changing vegetation demands on soil water. Applied to Austria over the past half-century the BIOME-BGC indices show a nationwide downward trend in days of no fire risk in both summer and winter, a reduction in days of low fire risk in summer, and an increase in extreme fire days in summer in most regions.

## Figures and Tables

**Fig. S1:**
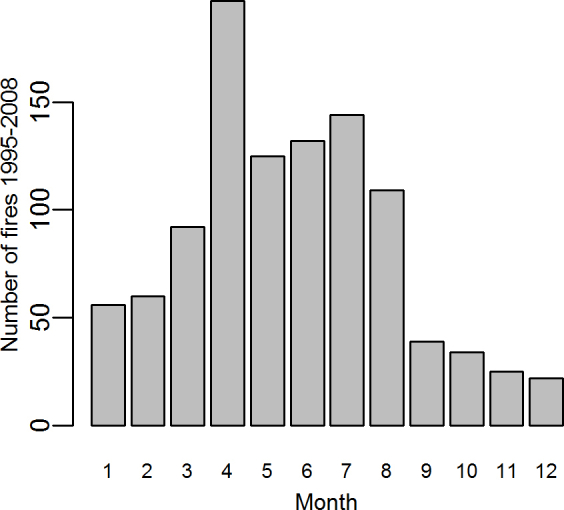
Bimodal monthly distribution of forest fire records in the Austrian wildfire database throughout the years in the period 1995–2008.

**Fig. S2:**
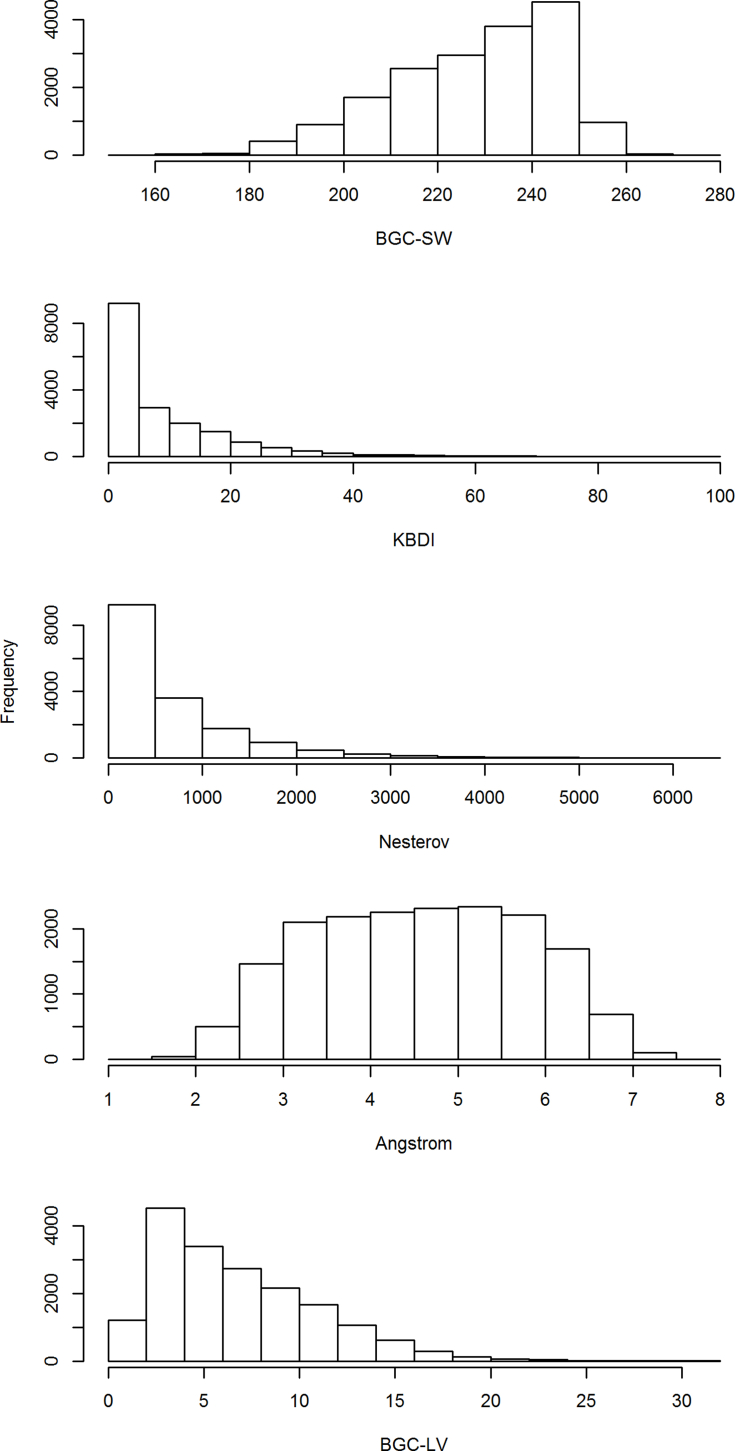
Frequency of occurrence histograms for the five indices, showing the distribution of daily values from 1960 to 2008. In the BGC-SW and Angström indices higher values indicate lower fire ignition risk, in the others higher.

**Table 1:** Risk classes of the Nesterov index (Skvarenina et al., 2003).

Index range	Class	Description
<300	1	No fire risk
301–1000	2	Low fire risk
1001–4000	3	Medium fire risk
4001–10 000	4	High fire risk
>10 000	5	Very high fire risk

**Table 2:** Index skill scores. Best result in bold, worst in italic.

Season		BGC-SW	KBDI	Nesterov	Angström	BGC-LV
Winter (150 days)	Intercept^a^	*3.219185*	30.04338	45.48407	46.74641	**52.77944**
	Slope^a^	*0.380119*	0.297503	0.254389	0.238598	**0.207937**
	Extremes^b^	**6**	**6**	**6**	*3*	**6**
Summer (215 days)	Intercept^a^	*26.91207*	30.78852	33.53935	40.08718	**46.27047**
	Slope^a^	*0.214572*	0.202856	0.188093	0.17453	**0.148444**
	Extremes^b^	6	**7**	5	*4*	*4*

a Intercept and slope of the ranked percentile plot (Eastaugh et al., 2012, see Fig. S3 in the supplementary information).

b Number of days where fires detected with an index value above the 99.67th percentile (winter) or 99.77th percentile (summer).

**Table 3:** Class limits for the BGC-LV index.

Nesterov lower bound	Percentile	Class	BGC-LV lower bound	BGC-LV upper bound	Description
0		1	0	5.32	No fire risk
300	45.25	2	5.33	10.22	Low fire risk
1000	79.95	3	10.23	20.57	Medium fire risk
4000	99.49	4	20.58	39.99	High fire risk
10 000		5	40		Very high fire risk

**Table 4:** Trends in summer fire ignition risk in each climate change region. Trends stronger than the ‘0’ region are shown in bold.

Region	Class 1		Class 2		Class 3		Class 4		Class 5	
	slope	sig^a^	slope	sig	slope	sig	slope	sig	slope	sig
0	−0.59112	***	−0.65908	**	0.799388	**	0.450816	***	0	ns
1	**−0.06**	ns	−0.18153	ns	0.17949	ns	0.062041	ns	0	ns
2	**−0.86847**	***	−0.47531	***	**1.058367**	***	0.285408	***	0	ns
3	−0.52102	***	−0.30745	*	0.506122	**	0.322347	***	0	ns
4	**−0.68449**	***	−0.48531	**	0.535714	**	**0.631939**	***	**0.002143**	ns
5	**−0.71347**	**	−0.10633	ns	0.703265	**	0.116531	**	0	ns
6	**−0.91929**	***	−0.26224	.	**0.815306**	***	0.366224	***	0	ns
7	**−0.85214**	***	**−0.69684**	***	0.692857	***	**0.832857**	***	**0.023265**	.
8	**−0.66704**	***	**−0.68878**	**	**0.824592**	**	**0.529388**	***	**0.001837**	ns
9	−0.49	**	−0.48541	*	0.678673	*	0.293163	**	**0.003571**	ns

^a^Significance levels: *p* value ‘ns’ > 0.1 > ‘.’ > 0.05 > ‘*’ > 0.01 > ‘**’ > 0.001 > ‘***’.

**Table 5:** Trends in winter fire ignition risk in each climate change region. Trends stronger than the ‘0’ region are shown in bold.

Region	Class 1		Class 2		Class 3		Class 4		Class 5	
	slope	sig^a^	slope	sig	slope	sig	slope	sig	slope	sig
0	−0.77439	***	0.460204	***	0.289592	**	0.024592	.	0	ns
1	−0.30755	*	0.222857	*	0.084694	.	0	ns	0	ns
2	−0.66551	***	**0.529184**	***	0.136327	**	0	ns	0	ns
3	−0.50439	**	0.314694	**	0.183367	*	0.006327	*	0	ns
4	−0.46112	**	0.253469	**	0.191429	**	0.016224	*	0	ns
5	−0.25867	**	0.194592	*	0.064082	.	0	ns	0	ns
6	−0.70286	***	0.510612	***	0.186327	**	0.005918	ns	0	ns
7	**−1.1549**	***	0.703673	***	**0.380612**	***	**0.070612**	*	0	ns
8	**−0.82724**	***	0.429286	***	**0.388776**	***	0.009184	ns	0	ns
9	**−0.90153**	***	**0.461122**	***	**0.389796**	**	**0.050612**	*	0	ns

^a^Significance levels: *p* value ‘ns’ > 0.1 > ‘.’ > 0.05 > ‘*’ > 0.01 > ‘**’ > 0.001 > ‘***’.

## Appendix A Supplementary data

Table S1 Ecophysiological parameters for BIOME-BGC modelling.
